# A Population-Based Conceptual Framework for Evaluating the Role of Healthcare Services in Place of Death

**DOI:** 10.3390/healthcare6030107

**Published:** 2018-08-30

**Authors:** Wei Gao, Sumaya Huque, Myfanwy Morgan, Irene J. Higginson

**Affiliations:** 1 Cicely Saunders Institute of Palliative Care, Policy and Rehabilitation, King’s College London, Bessemer Road, Denmark Hill, London SE5 9PJ, UK; Sumaya.huque@gmail.com (S.H.); Irene.higginson@kcl.ac.uk (I.J.H.); 2 Institute of Pharmaceutical Science, King’s College London, London SE1 9NH, UK; Myfanwy.morgan@kcl.ac.uk

**Keywords:** place of death, healthcare services, conceptual framework, end of life care outcome, end of life care policies and commissioning, determinants

## Abstract

Background: There is a significant geographical disparity in place of death. Socio-demographic and disease-related variables only explain less than a quarter of the variation. Healthcare service factors may account for some (or much) of the remaining variation but their effects have never been systematically evaluated, partly due to the lack of a conceptual framework. This study aims to propose a population-based framework to guide the evaluation of the role of the healthcare service factors in place of death. Methods: Review and synthesis of health service models that include the impact of a service component on either place of death/end of life care outcomes or service access/utilization. Results: The framework conceptualizes the impact of healthcare services on the place of death as starting from the end of life care policies that in turn influence service commissioning and shape healthcare service characteristics, including service type, service capacity—facilities, service location, and workforce, through which service utilization and ultimately place of death are affected. Patient socio-demographics, disease-related variables, family and community support and social care also influence place of death, but they are not the focus of this framework and therefore are grouped as needs and other environmental factors. Information on service utilization, together with the place of death, creates loop feedback to inform policy and service commission. Conclusions: The framework provides guidance for analysis aiming to understand the role of healthcare services in place of death. It aids the interpretation of results in the light of existing knowledge and potentially identifies service factors that can be addressed to improve end of life care.

## 1. Background

Place of death has evolved over the past two decades from a quality indicator to an outcome measure in end of life care (EoLC) [1,2]. Although the majority of terminally ill patients prefer to die at home or in a home-like environment such as hospices, hospitals remain the most common place of death. In 2001–2010, of all deaths from non-accidental causes (*N* = 4.6 million) in England, 57% occurred in a hospital, 19% at home, 17% in a care home, and only 5% in hospice [3]. It is a national commitment of the United Kingdom (UK) policy to offer people who are approaching the end of life to die in a place of their choice [4,5] with national and local efforts directed to facilitating such a choice. However, without high-quality EoLC provision, the choice of place of death can never be a real one. A survey of 245 family physicians found that 94% of patients admitted to hospital with limited life expectancy was due to an inadequate care provision (i.e., an acute situation for which the care setting was not prepared) in their usual care setting [6]. Characterizing where people die and the factors that influence their site of death is important to inform the development and implementation of policy and EoLC services.

A systematic review [7] involving 58 studies, with over 1.5 million patients from 13 countries, concluded that place of death results from interactions between three main groups of factors: those related to the illness, the individual, and the environment. The GUIDE_Care project which investigated variations in place of death using routine death registry data found that individual patient level characteristics, such as age, marital status and diagnosis, were able to explain only a quarter of the variation in place of death [3]. These findings suggest that variables related to healthcare services may have a role in determining the place of death. However, there is a scarcity of empirical studies that have systematically assessed the influence of healthcare service provision on place of death. Two major reviews [7,8] on the determinants of place of death identified a total of 87 studies, few of which evaluated the role of healthcare services in place of death. A key barrier, as identified by Phillips et al., is the lack of a conceptual model [9].

The aim of this paper is to propose a conceptual framework to guide the planning, analysis, and interpretation of factors related to healthcare services that may influence place of death.

## 2. Methods

The development of this framework was built on a conceptual model of factors influencing death at home by Gomes & Higginson [7], identified through the search of published and peer-reviewed literatures (up to August 2018) in Ovid MEDLINE, EMBASE and PsycInfo. The details of the search are enclosed in Appendix A. It was also the only theoretical model on the determinants of place of death resulting from our literature search. However, this model was developed from empirical studies and contains limited information on healthcare variables. Hence, we identified six further health service models, which include the impact of a service component on either end of life care outcomes or service access/utilization [9,10,11,12,13,14]. Other than Laguna’s model, which was selected on the basis of its close relevance to end of life care, the other models were generic but widely cited in health service research. The service components that may potentially influence place of death (Table 1) and their sequential organization were identified from and guided by these models.

## 3. Results

### 3.1. The Conceptual Framework

**Figure 1:**
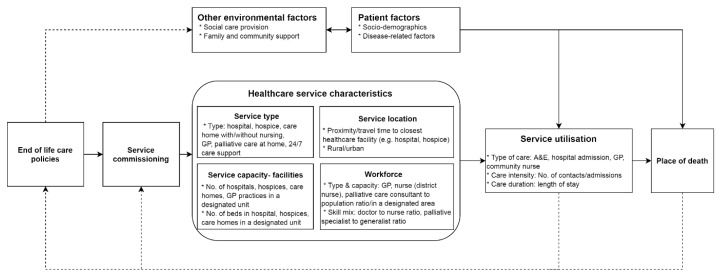
A conceptual framework for the role of healthcare service factors in place of death.

Healthcare services in this framework refer to all health and care services related to end of life care; these include generic (e.g., hospital, general practice) and specialized (e.g., hospice) care services (Figure 1). The characteristics of such provision initially depend on the EoLC policies and their implementation through healthcare service commissioning, which, in turn, influences service utilization and ultimately where people die. Individual socio-demographic and disease-related characteristics (patient factors), together with social care, and family and community support (environmental factors), are not the focus of this framework; they are included as the variables to be controlled for when evaluating the service impact on the place of death. Information on service utilization and place of death create loop feedback to inform end of life care policies and service commissioning. The arrows indicate the direction of the impact. The solid and dotted lines represent direct and indirect effects (or feedback loop), respectively.

### 3.2. End of Life Care Policies

Variations in health care utilization and outcomes between geographic areas have been linked to broad health system policies and provision [15], although no formal research has evaluated the policy impact on the population-based end of life care outcomes. However, a series of large-scale natural experimental studies using the English death registry database provided some evidence for a causal link between national EoLC improvement efforts, which promote community based care and increased home deaths [2,16,17,18]. The addition of EoLC policies in our framework prompts the identification of concrete actions and policy levers that have been taken to improve EoLC quality [4].

### 3.3. Service Commissioning

Healthcare service planning and commissioning is the process of deciding what kinds of services should be provided to local populations, who should provide them, and how they should be paid for. The UK’s End of Life Care Strategy (2008) focused on raising the profile of EoLC with strategic commissioning, delivery of high-quality services, and enhanced education, training and research in the field [4]. The strategy also emphasizes the importance of coordinated care and support for caregivers. This is supported by evidence from a study in the United States that spending on home and community-based services increases the chance of home death, through reducing the risk of the end of life relocation to a nursing home [19].

### 3.4. Healthcare Service Characteristics

Healthcare services are characterized by type, capacity (facilities and workforces) and location. These characteristics can be perceived as resulting from EoLC policies requiring service commissioning and service providers to take actions to improve EoLC and its outcomes. We put these characteristics in separate boxes, signifying that they are relatively independent of each other and cite some indicators that may be used to represent the corresponding service characteristics.

### 3.5. Service Type

The service options available to patients is a key determinant of where they die [7,8]. When hospital-based care dominated the healthcare system, most people died in hospitals [20]. However place of death has been slowly but steadily shifting to home or usual residence over the past two decades in countries with efforts to promote community-based palliative and end of life care [3,18,20,21,22,23,24]. Studies have shown that home-based care delivered at a patient’s home is effective in enabling home deaths [25,26]. General practitioners’ home visits during the last three months was positively associated with death at home [27]. Enrolment in palliative care programs was associated with lower odds of dying in hospitals [28]. For analysis purposes, type of care can be categorized into primary (General Practice—GP, care home), secondary (hospital) and tertiary (specialist palliative services) care.

### 3.6. Service Capacity—Facilities

A key component of the healthcare system is the resources (capital and labor) devoted to healthcare [10]. The mere presence of a certain type of service is insufficient to lead to the use of the service and a change in place of death with the need for an appropriate fit between the population’s demand and the supplies of the healthcare delivery system. The European Association for Palliative Care (EAPC) recommended that the optimal level of in-patient palliative care provision is 80–100 bed per million population. This estimation takes into account the needs for both cancer and non-cancer conditions [29]. A 10-year large-scale retrospective cohort study of patients with lung cancer found that hospice death was more likely in areas with more hospice beds [30]. Similar supply-side (service provision and availability) effects were also seen in the pattern of hospice deaths in children and young people with cancer—overall, hospice death was low but more than doubled in the proportion from 6% to 13% during the study period, where hospice service provision was also improved [18]. Rates (number of facilities, beds in a defined unit, e.g., per 100,000 inhabitants) and densities (number of facilities, beds or staffing in a defined size of the area, e.g., per square kilometers) can be used to measure and compare service capacity of different regions.

### 3.7. Service Location

A growing body of research suggests that the geographical accessibility of healthcare facilities, and not merely the general level of provision of services in a geographic area, has a definitive role in service utilization and health outcomes [11,31,32]. Proximity to a specific healthcare facility, (e.g., hospital, hospice or nursing home) has been shown to increase the probability of cancer patients dying in that particular facility [33]. The geographical location of services has implication for resource allocation and optimization. We, therefore, included the location of healthcare services as a determinant of the place of death in our conceptual model. Geographical accessibility to services can be measured at the individual or area level. A commonly used measure is the distance from a patient’s residential address to their closest hospital, hospice, and care home [34], or the average of the individually measured distances of a group of patients in an area of interests for an area level measure. With technology advancement, more sophisticated measures (e.g., travel time incorporating road attributes) have been proposed and developed [35]. Rural/urban settlement, which is often identified as an attributable factor for disparities in service usage and place of death [36,37], can also be used as a proxy measure for service availability and accessibility.

### 3.8. Workforce

Healthcare services can be viewed as complex and interdependent interventions that lead to changes in health and care outcomes. Delivering high-quality EoLC requires a well-developed and highly competent workforce [38,39]. The involvement of general practitioners in facilitating patients to achieve their preferred place of death is consistent across studies [27,40,41]. The mechanism through which the workforce exerts an impact on the place of death is yet unknown but may be related to the following aspects: the category of the workforce (e.g., general practitioner, nurse, palliative care consultant), staffing level, and skill mix. The staffing level can be measured by the number of full-time equivalent employees, such as the nurse-to-patient ratio [42].

Skill mix refers to the mix and breadth of staff, professions, and experience and/or qualifications [43]. The right skill mix is needed for both effective and efficient patient care, including the end of life care [44]; it is a quality statement (QS16) in the National Institute for Health and Care Excellence (NICE) guidance for End of Life Care for adults [45]. The EAPC recommended that the core team for palliative care should consist of doctors and nurses as a minimum [46], though no study has investigated whether and how skill mix is related to place of death. This needs to be addressed in future studies. A common measure for the skill mix is the ratio of the number of staff in selected (usually two) workforce categories. For example, palliative care consultant to nurse ratio.

As palliative and end of life care is a multidisciplinary approach encompassing not only physical but also psychological, social and spiritual components, future workforce evaluation studies should also consider non-healthcare workforces.

### 3.9. Service Utilisation

It is through utilizing service that the healthcare delivery system achieves its impact on the place of death. Service utilization can be measured at area level by the number of admissions, lengths of stays, and bed occupancies in hospitals, hospices, and emergency units. Ecological level (e.g., national, area) statistics on health service use are routinely released to the public domain [47,48,49,50]. These data are often summarised by care settings though that which are specific to PEoLC are very limited. The National End of Life Care Intelligence Network of Public Health England (http://www.endoflifecare-intelligence.org.uk) has been set up to fill this information gap but is still well-behind other care settings. According to our framework, service utilization should be modelled as a mediating factor between service characteristics and place of death outcome [51].

### 3.10. Needs and Other Environmental Factors

The focus of this framework is the healthcare service input on the place of death. Therefore, we group all other non-health service variables that may influence place of death as needs and other environmental factors. Needs factors include the clinical and socio-demographic characteristics of individuals [7,52]. The provision of PEoLC was historically focused on patients with terminal cancer. However, the modern view is that PEoLC services should be provided on the basis of needs rather than the diagnosis. Patients with certain non-cancer conditions, frail older people, and patients with multiple morbidities may have PEoLC needs and can benefit from palliative care input [53]. One method of measuring service needs at the population level can be using underlying (e.g., cancer versus non-cancer) and contributory causes of deaths (e.g., the number of comorbidities) reported in routine death registry data [54].

Family and community support are crucial in enabling dying people to remain at home or in their usual residence. Marital status is often used as a proxy for family support. A national population-based study in cancer patients in England found patients who are single, divorced, or widowed were less likely to die at home, compared to those who are married [2]. At the population level, the percentage of individuals summarised according to their marital status is freely available through official statistics websites, e.g., the Office for National Statistics (ONS)’census portal [55]. Area-level spending on social care may serve as an indicator of the level of social care provision. There has not yet been a formal evaluation of the impact of social care provision on place of death, but social care (measured at the individual level) costs were negatively associated with inpatient care costs [56], suggesting social care may play a role in place of death. A recent Australian study provided further qualitative evidence that specially trained community care workers can effectively support patients and their families in home settings at the end of life [57], though none of them have quantified the effects of social care.

## 4. Discussion

We present a conceptual framework identifying service factors that may affect the place of death and how. To our knowledge, this is the first-of-its-kind for an important end of life care outcome—place of death. This model can be used to guide the evaluation of the population-based impact of service factors on place of death, from data collection to data analysis and interpretation. To date, the majority of published studies on the place of death has a focus on individual socio-demographic and clinical characteristics [2,16,58,59]. A large-scale observational study conducted in the UK found that only a quarter, or even less for certain diseases, of the geographical variation in place of death are explained by patient-level characteristics [3].

The framework notes the importance of various service components in determining where people die; they are positioned in the middle of a causal pathway. The chain of reaction starts from the national policy followed by service commissioning. They go on to affect service characteristics, which then influence service utilization and ultimately place of death. The framework can be used to guide population-based service planning and development. Service commissioners should take into consideration four aspects of service factors: type, capacity (facilities), location, and workforce. The relative contribution of the individual components to service utilization and place of death has not yet been quantified, but it is critical to help design programs and prioritize intervention. It is worth noting that the feedback loop between service utilization, place of death, and policies that will change service characteristics through planning and commissioning, highlighting intervention at any levels (e.g., policy, planning and commissioning, service), should be a dynamic rather than a one-off process of learning.

A patient’s palliative care needs encompass four dimensions: physical, psychological, social, and spiritual. According to Maslow [60], these needs are in hierarchical structures with physical at the lowest and spiritual the highest level. It means that the higher needs may not be relevant until the lower ones are met. Therefore, unless we can provide high-quality end of life care to meet a patient’s lower level (e.g., physical) needs across all available settings, the preferred place of care and death do not have a real meaning to patients and families. In fact, patients choose a hospital as their preferred setting for the end of life care because they feel that the hospital is safer than other care settings, e.g., their own home or usual residence [61]. In other words, patients do not always feel safe at home or that care need can be best met at home—this, per se, indicates service gaps. 

The key challenge for population-based service evaluation is data availability. The data scoping exercise for the GUIDE_Care Services project identified large gaps in service data. There exists very limited national-level service data in palliative and end of life care. Even a master list of hospices was not available in the public domain with easy access options to researchers and stakeholders. This is in contrast to personal-level data, where the major challenge lies in accessibility issues related to information governance, confidentiality, and data protection [62]. Service data are collected at the aggregate level and do not require ethical approvals for access. Even for commissioning data, which is supposed to be open and transparent, there is no centralized data portal. In England, only an annual CCG spending of over 25 k on palliative and end of life care is available in the public domain; however, not all CCGs submit this data. Service data collection is not as well developed as that of person-level data; most of the service-level data collections take place at the local level and a few at a larger scale or national level. The methods for local data collection vary widely, and data reporting and capture are far from being a routine at all levels. These all hamper the effort to evaluate and understand the role of service factors in place of death. There is urgent need to develop systems for service data collection.

As service variables are collected at the aggregate level, the choice of analytical methods is dependent on the study unit. If it is at service level, then the data can be analyzed using conventional statistical methods, which assume the independence of units of analysis. For studies with patients as the unit of analysis and involving a mixture of service- and individual-level variables, more sophisticated analytical methods such as multilevel models or more appropriately causal modelling should be used to account for the hierarchical structure of the data and the complex relationship between the variables at various levels.

We did not include a quality aspect (e.g., the quality of service delivery) for it is hard to measure, particularly at the population level. Even in a research context, it is challenging to ensure that healthcare service is delivered as intended [63]. Therefore, when applying the framework, one should bear in mind that the service characteristics may not be fully reflected in the variables collected for analysis. One should also note that the major building blocks of our framework were drawn from robust theoretical models and comprehensive systematic reviews, based primarily on studies undertaken in the UK, western European countries and the USA, and therefore they may not be relevant to other healthcare systems. Finally, as the place of death reflects only the last moments of a person’s life, it should be viewed in conjunction with the other end of life care outcome measures, for example, place of care and care transitions [24]. This will provide a more comprehensive perspective about how patients are cared for at the end of life.

Currently, there are a limited number of studies investigating the service characteristics that are most amenable to change. We hope our proposed framework will facilitate research efforts on the place of death to include more healthcare service factors.

## 5. Conclusions

We propose a framework which conceptualizes the impact of healthcare services on the place of death. The impact pathway is proceeding from the end of life care policies, which, in turn, influence service commissioning and shape healthcare service characteristics, including service type, capacity (facilities), location and workforce, through which service utilization and ultimately place of death are affected. Patient socio-demographics, disease-related variables, and social care and support related factors also influence place of death, but they are not the focus of this framework; they are therefore grouped as needs and other environmental factors. Information on service utilization together with place of death outcome create a loop feedback to inform policy and service commissioning. The framework can be used to guide the planning, analysis, and interpretation of the service-related factors that may influence place of death. It can also be used to potentially identify service factors that may be addressed to improve end of life care.

## Figures and Tables

**Table 1:** The components of the referenced models contributed.

Models	Contributed Components
Gomes & Higginson, 2006	Patient factors, other environmental factors
Phillips, et al., 1998	Policies, commissioning
Andersen & Newman, 2005	Service type, service capacity—facilities
Levesque et al., 2013	Service location, commissioning
Donabedian, 1988	Workforce
Kindig et al., 2008	Commissioning, service location
Laguna et al., 2012	Service type, workforce, commissioning

## Appendix A. Details of the Literature Search

Aim:

To identify the published conceptual framework on the determinants of place of death.

Search databases:

1. Embase 1974 to 2018 Week 34
2. Ovid MEDLINE(R) and Epub Ahead of Print, In-Process & Other Non-Indexed Citations and Daily 1946 to August 22, 2018
3. PsycINFO 1806 to August Week 3 2018

Search strategy:

(Palliative care or end of life care or hospice care or terminal care or death or dying or terminally ill or palliative nursing) AND (conceptual framework or conceptual model or pragmatic framework or pragmatic model or theoretical model or theoretical framework) AND (site of death or place of death or home death or hospital death or hospice death or nursing home death or care home death) in all searchable fields in the three databases. Duplicated records were deleted.

Result:

Only one conceptual model (Gomes & Higginson, 2006) was identified.
